# Human Cytomegalovirus Infection and Breast Cancer: A Literature Review of Clinical and Experimental Data

**DOI:** 10.3390/biology14020174

**Published:** 2025-02-08

**Authors:** Rancés Blanco, Juan P. Muñoz

**Affiliations:** 1 Independent Researcher, La Florida 8240000, Chile; 2 Laboratorio de Bioquímica, Departamento de Química, Facultad de Ciencias, Universidad de Tarapacá, Arica 1000007, Chile

**Keywords:** breast cancer, human cytomegalovirus, HCMV, oncoproteins, viral oncogenesis, risk factors

## Abstract

Breast cancer (BC) remains a significant global health challenge, highlighting the need for continued research into novel risk factors, diagnostic approaches, and personalized treatments. Among emerging risk factors, viral infections have been implicated as potential contributors to breast carcinogenesis and BC progression. Recent evidence suggests that specific oncogenic strains of human cytomegalovirus (HCMV) may have the capacity to transform human mammary epithelial cells. This review assesses clinical data regarding HCMV presence in both tumor and non-tumor breast tissues, examining the role of HCMV oncoproteins in BC development and progression. Current findings indicate a higher prevalence of HCMV infection in breast carcinomas compared to non-tumor tissues, associated with an elevated risk of BC. Additionally, the HCMV-driven breast carcinogenesis model proposed here suggests that HCMV oncoproteins may activate multiple oncogenic pathways, fostering cell proliferation, survival, and tumor development. A deeper understanding of the role of HCMV in BC could enhance risk stratification and support the creation of targeted therapeutic strategies.

## Simple Summary

Human cytomegalovirus (HCMV) has been suggested as a potential contributor to breast cancer (BC) development, though its role remains the subject of ongoing debate. This review examines the epidemiological evidence linking HCMV infection to BC and explores the molecular mechanisms by which the virus may contribute to cancer initiation and progression. The current literature reveals a higher prevalence of HCMV in BC tissues compared to normal or benign breast tissues. Furthermore, in vitro studies suggest that HCMV can activate key pathways involved in tumor growth, survival, and metastasis. The virus may also influence the tumor microenvironment and immune responses, fostering conditions that promote cancer progression. Finally, the evidence indicates that certain HCMV strains possess oncogenic properties and may be associated with more aggressive BC subtypes. However, further research is necessary to confirm these findings and translate them into practical clinical applications.

## 1. Breast Cancer (BC) as a Major Global Health Problem

### 1.1. The Epidemiology of BC

BC is the most frequently diagnosed malignant neoplasm and the leading cause of cancer-related mortality among women worldwide. In 2022, BC accounted for 2,308,897 new cases and 665,684 deaths globally, representing 11.6% of all cancer incidences and 6.9% of cancer-related deaths [1]. The risk of BC development varies by country and ethnicity due to differences in risk factor exposure and the availability of primary prevention programs [2]. The highest BC incidence rates are reported in regions such as France, Australia/New Zealand, Northern Europe, and North America, where rates are nearly four times higher than those in South-Central Asia and Middle Africa [2].

Although women in developed countries have higher BC incidence rates (54.1 vs. 30.8 per 100,000) than those in transitioning economies, they experience lower mortality rates (11.3 vs. 15.3 per 100,000). By contrast, regions like Melanesia, Western Africa, and Micronesia/Polynesia report the highest BC mortality rates [1]. Increased mortality rates in transitioning countries may result from limited healthcare access and infrastructure, which often lead to diagnostic delays and the presentation of disease at later stages [3,4].

Tumor recurrence and metastasis are the primary contributors to BC-related deaths. Despite advancements in the early detection and treatment of primary breast tumors, approximately 20–40% of BC patients experience recurrence. Recurrence rates are influenced by factors including the stage at diagnosis, histological grade, and molecular classification of the tumor [5,6,7].

### 1.2. Histological and Molecular Classification of BC

BC encompasses a heterogeneous group of tumors originating from epithelial cells lining the breast milk ducts or the cells of milk-producing glands. Histologically, most BC cases are adenocarcinomas (90–95%), of which 75–80% are invasive ductal carcinomas (IDC), 5–10% are invasive lobular carcinomas (ILC), and 4–6% are mixed carcinomas (IDC/ILC) [8]. Less common types of invasive BC include tubular, cribriform, mucinous, medullary, pleomorphic, papillary, apocrine, metaplastic, and adenoid cystic carcinomas, among others [8,9].

BC tumors are classified by their degree of differentiation—based on tubule/gland formation, nuclear pleomorphism, and mitotic count—into three grades: well-differentiated (grade I), moderately differentiated (grade II), and poorly differentiated (grade III) [10]. The prognostic value of both histological classification and grading has been well-documented. For example, Wasif et al. (2010) reported a poorer prognosis for patients with IDC compared to ILC when matched by stage [11]. Similarly, Rakha et al. (2008) demonstrated a correlation between histological grade and cancer-specific survival in BC patients [12]. However, increasing evidence indicates that morphological parameters alone are insufficient to fully capture the biological heterogeneity of BC. This recognition has led to the integration of molecular markers, which provide both prognostic and predictive insights [13].

The expression of hormone receptors (HR), including estrogen receptor (ER) and progesterone receptor (PR), as well as human epidermal growth factor receptor 2 (HER2), is pivotal in BC development [14,15]. Based on the immunohistochemical detection of these receptors, BC is categorized into four molecular subtypes: Luminal-like (A and B), HER2-enriched, and triple-negative BC (TNBC) [16]. Additionally, the Ki-67 index helps distinguish Luminal A from Luminal B subtypes [17]. Luminal A tumors, representing 60% of BC cases, are ER and/or PR positive and HER2-negative, with a Ki-67 index below 14% [18]. These tumors generally have a low histological grade and are associated with a favorable prognosis compared to other BC subtypes [19].

The Luminal B subtype comprises 15–20% of BC cases and is defined by ER and/or PR positivity, with HER2 either positive or negative, and a Ki-67 index above 14% [18]. Luminal B tumors exhibit a more aggressive phenotype, higher histological grade, greater recurrence rate, and worse prognosis than Luminal A tumors [20,21]. The HER2-enriched subtype, accounting for approximately 20% of BC cases, includes HR-negative, HER2-positive tumors with a HER2 immunohistochemical score of 3+ or 2+ and confirmed gene amplification by in situ hybridization (ISH) [22]. HER2 plays a key role in promoting BC cell proliferation, differentiation, and survival [15], leading to an aggressive biological profile and poor prognosis associated with HER2-enriched BCs [15].

TNBC is a heterogeneous subtype characterized by the absence of HR and HER2 (ER-/PR-/HER2-) and comprising 10–20% of BC cases [8]. It is generally associated with the poorest clinical outcomes among BC subtypes, exhibiting high recurrence and metastasis rates and lacking specific therapeutic targets [23]. TNBC can be further divided into various subtypes based on gene expression profiles, including basal-like (BL1 and BL2), claudin-low, immunomodulatory, mesenchymal-like, mesenchymal stem-like, and luminal androgen receptor subtypes [24,25]. Among these, the basal-like subtype is the most prevalent, marked by the expression of high-molecular-weight cytokeratins (CK5/6, CK14, CK17) common to basal/myoepithelial cells of normal breast tissue and also expressing the epidermal growth factor receptor (EGFR) [26]. Basal-like BC is often associated with *BRCA1* gene mutations, which are a significant risk factor for familial BC [27].

### 1.3. Risk Factors Associated to BC Development

A range of risk factors contributes to BC development, including age, reproductive history, estrogen levels, lifestyle, family history, and genetic mutations. However, BC is most likely influenced by a combination of genetic, environmental, and lifestyle factors [28]. For example, parity has been linked to an increased risk of basal-like BC, with odds ratios (OR) of 1.8 for those with 1–2 children (95% CI: 1.1–1.3) and 1.9 for those with three or more children (95% CI: 1.1–3.3) compared to nulliparous women [29]. Additionally, never breastfeeding was associated with a higher risk of basal-like BC (OR = 4.17, 95% CI: 1.89–9.21) compared to women who were either nulliparous or had breastfed [30].

Among postmenopausal women who did not use menopausal hormone therapy (MHT), those with three to five lifestyle risk factors showed an increased risk of ER-positive BC (HR = 2.42, 95% CI: 1.27–4.63) relative to women with no lifestyle risk factors [31]. Alcohol and tobacco use have also been implicated in BC development [32]; for instance, the relative risk (RR) of BC was 1.32 (95% CI: 1.19–1.45) for women consuming 44–55 mL of alcohol per day and 1.46 (95% CI: 1.33–1.61) for those consuming 57 mL or more per day [33]. Furthermore, an elevated risk of contralateral BC was observed among women who consumed alcohol and smoked post-diagnosis (RR = 1.62, 95% CI: 1.24–2.11) [34]. A meta-analysis by Liu et al. (2021) confirmed a strong association between family history of BC and increased BC risk, with an OR of 2.02 (95% CI: 1.83–2.23; *p* < 0.00001) [35].

On the other hand, some germline mutations are significantly associated with an elevated risk of BC development. Among these, *BRCA1* (located at 17q21.31) and *BRCA2* (located at 13q13.1) are the primary high-penetrance genes involved [36]. Both *BRCA1* and *BRCA2* are tumor suppressor genes that play critical roles in repairing double-stranded DNA breaks through homologous recombination [37]. A meta-analysis by Chen and Parmigiani (2007) reported cumulative BC risks in *BRCA1*- and *BRCA2*-mutation carriers over the age of 70 as 57% and 49%, respectively [38]. Notably, protein-truncating variants in *BRCA1* (OR = 10.57, 95% CI: 8.02–13.93; *p* < 0.0001) and *BRCA2* (OR = 5.85, 95% CI: 4.85–7.06; *p* < 0.0001) were strongly associated with increased BC risk [39].

Other high-penetrance genes linked to BC include *TP53*, *CDH1*, *PTEN*, and *STK11* [40,41,42,43]. Additionally, DNA repair genes related to *BRCA*, such as *ATM*, *CHEK2*, PALB2, and *BRIP1*, confer an intermediate risk of BC [44,45,46,47]. A study by the BC Association Consortium (2021) found that protein-truncating variants in *ATM* (OR = 2.10, 95% CI: 1.71–2.57; *p* < 0.0001) and *CHEK2* (OR = 2.54, 95% CI: 2.21–2.91; *p* < 0.0001) were associated with a 2.0- to 2.5-fold increased risk of BC [39]. Variants in genes like *BARD1*, *RAD51C*, *RAD51D*, and *MSH6* were also moderately associated with BC risk, with ORs ranging from 2.09 to 1.96 [39].

In summary, approximately 25% of BC cases are attributed to inherited mutations, while the majority (70–75%) are considered sporadic [48]. Sporadic BC develops through a multistep process involving the accumulation of somatic mutations in critical cellular pathways, with no germline mutations contributing to carcinogenesis [49]. Key oncogenes involved in sporadic BC include *TP53*, *RB*, *MYC*, *CCND1*, *Bax*, *Bcl2*, *CDH1*, HRs, and *ERBB2* (HER2/neu) [49]. Interestingly, some of these genes are targets for oncogenic viruses that may play a role in BC development.

## 2. HCMV Infection in BC

### 2.1. Viral Infections as Risk Factors for BC

Viral infections are estimated to account for 15–20% of all human malignancies [50]. Growing evidence points to a role for oncogenic viruses in the malignant transformation of mammary epithelial cells and the development of BC [51]. Viruses implicated in this process include mouse mammary tumor virus (MMTV), high-risk human papillomavirus (HR-HPV), Epstein–Barr virus (EBV), bovine leukemia virus (BLV), and human cytomegalovirus (HCMV) [52].

MMTV has been detected in 26% (1320/5015) of BC cases, with higher prevalence in regions like North Africa and Australia (40%) compared to North America, South America, Europe, and Asia, where rates range from 18–28% [53]. A review by Lawson and Glenn (2022) analyzed 26 case-control studies from various laboratories, finding MMTV in approximately 35% of BC samples compared to less than 2% in control breast tissue [54]. Furthermore, MMTV was detected by Fluorescence-nested PCR in 30.3% of sporadic BC cases (17 of 56) and only in 4.2% of hereditary BC (2 of 47), suggesting an association between MMTV and sporadic BC [55]. However, some studies did not detect MMTV in BC samples, possibly due to the low viral load in BC tissues and the challenges in detecting MMTV sequences [54,56,57].

HR-HPV has also been proposed as a factor in BC development. A meta-analysis by Ren et al. (2019) encompassing 37 case-control studies with 3607 BC and 1728 non-malignant breast tissues found a six-fold increase in BC risk in the presence of HPV (OR 6.22, 95% CI: 4.25–9.12; *p* = 0.0002). This association remained significant for specific HPV subtypes, including HPV16, HPV18, and HPV33 [58]. Another meta-analysis of nine case-control studies further supported the link between HPV and BC (OR 5.9; 95% CI: 3.26–10.67) [59]. Increased HPV DNA presence has been noted in TNBC cases compared to other BC subtypes [60,61,62]. Conversely, other studies have reported an absence of HPV DNA in BC tissues, highlighting the mixed findings in this area [63,64]. 

EBV is another oncogenic virus implicated in the development of some malignancies, including undifferentiated nasopharyngeal carcinoma (NPC) and gastric cancer [65]. Farahmand et al. (2019) reported an overall prevalence of EBV infection in 26.37% (95% CI: 22–31%) of BC patients, based on a study of 4607 cases from 26 countries. A meta-analysis of 30 case-control studies further indicated an association between EBV infection and increased BC risk (OR 4.74, CI: 2.92–7.69; *p* < 0.0001) [66]. Additionally, EBV infection has been found to be more prevalent in hormone-receptor-negative BC, a more aggressive subtype [67]. However, some studies report an absence of EBV DNA in BC tissues, contributing to the ongoing debate on the role of EBV in BC [68,69,70]. These conflicting results may arise from variations in virus detection methods, epidemiological differences, and other variables [71].

BLV infection has also been found in BC samples [72,73]. Using in situ-PCR, Buehring et al. detected BLV DNA in 58.8% of BC samples (n = 114 cases), significantly higher than in controls (28.8%, n = 104) (OR 3.07, 95% CI: 1.66–5.69; *p* = 0.0004). BLV DNA was also present in 38% of premalignant breast tissues (n = 21 cases) [74]. Similarly, Baltzell et al. (2018) found a higher prevalence of the BLV tax DNA segment by ISH and PCR in BC samples compared to benign or normal breast tissues (OR 5.87; 95% CI: 2.83–12.16; *p* < 0.0001) and premalignant samples (OR 2.24; 95% CI: 1.03–4.84; *p* = 0.0572) [75]. Khan et al. detected BLV genes in 26.8% (728/2710) of BC samples versus 10% (10/80) of non-tumor tissues (*p* = 0.0029) through nested PCR [76]. A meta-analysis of nine case-control studies also showed an association between BLV infection and BC risk (OR 2.57; 95% CI: 1.45–4.56; *p* = 0.001) [72]. Despite these findings, the role of BLV as a risk factor remains under debate.

Finally, growing evidence suggests a potential role for high-risk HCMV (HR-HCMV) infection in initiating and advancing certain human malignancies, including BC [77,78]. Therefore, the association between HCMV infection and BC development will be further examined in the following sections.

### 2.2. Epidemiology of HCMV Infection in BC

HCMV, previously known as human herpesvirus-5 (HHV-5), is a widespread beta-herpesvirus infecting approximately 66–90% of the global population [79]. Typically, HCMV infections remain subclinical, likely due to the host immune system’s capacity to control viral replication [80]. In immunocompromised individuals, however, uncontrolled HCMV replication can lead to severe conditions, including pneumonitis, encephalitis, retinitis, hepatitis, esophagitis, and enterocolitis [81,82]. Recently, research has revisited the potential role of HCMV in human malignancies, including BC, owing to its ability to disrupt various cellular functions—such as cell cycle regulation, apoptosis, and DNA repair—induce chronic inflammation, and modulate antiviral immune responses [77,78].

HCMV DNA and/or proteins have been identified in various malignancies, including glioblastoma, prostate, colon, and ovarian adenocarcinomas, as well as in BC [83,84,85,86,87]. HCMV is known to infect diverse cell types, including epithelial cells, endothelial cells, fibroblasts, smooth muscle cells, leukocytes, and dendritic cells [88]. In this regard, studies concerning the detection of HCMV infection in breast tissues by PCR should be analyzed carefully, and other techniques such as ISH and immunohistochemistry (IHC) are needed to determine the lineage of HCMV-infected cells.

#### 2.2.1. HCMV Infection and Risk of BC Development

Several studies have demonstrated an elevated presence of HCMV in BC compared to normal or benign breast tissues, suggesting a potential role for HCMV in breast carcinogenesis [89,90,91,92]. These findings are summarized in Table 1 and Table 2. For example, HCMV DNA detected by in ISH was significantly more prevalent in BC samples (56.7%) compared to benign breast tumors (24.0%) and healthy breast tissues (0%) (*p* < 0.01) [89]. Similarly, other studies have shown a higher expression of HCMV proteins, such as immediate early (IE) antigens [87,93,94], late antigen (LA) [94], and pp65 [95], in BC samples relative to normal breast tissues.

Further supporting these findings, Cui et al. (2017) demonstrated HCMV *IE2* mRNA expression in 100% of BC tissues, statistically significant compared to surrounding non-cancerous tissues [96]. Importantly, the expression of HCMV IE1 (RR 8.9, CI: 3.04–26.33), LA (RR 56.35, CI: 3.68–83.84), and pp65 (RR 54.84, CI: 3.58–59.44) was associated with an increased risk of BC [97]. Some studies without control groups have also reported a high rate of HCMV in BC. For instance, Calderon et al. (2022) found HCMV in 72.5% of BC samples [98], and Ahmed and Yussif (2016) observed HCMV LA expression in 43.9% of BC cases using IHC [99]. Tsai et al. (2005) identified the HCMV IE2 sequence in 75.8% of BC tissues, though they found no significant differences compared to non-malignant samples [100]. Shadood et al. (2018) reported similar rates of HCMV DNA in both BC (6.25%) and normal tissues (5%) [101].

Conversely, some studies have detected low or minimal HCMV infection in BC. Utrera-Barillas et al. (2013) found HCMV DNA in only 7.4% of BC specimens [102], and Eghbali et al. (2012) detected HCMV DNA in only 8.3% of BC samples [103]. Other researchers have reported no evidence of HCMV DNA or proteins in BC tissues [104,105,106,107,108].

The variability in HCMV detection rates across studies presents a critical challenge in understanding the role of HCMV in breast carcinogenesis. This discrepancy could stem from methodological differences, including variations in the sensitivity and specificity of detection techniques such as ISH, IHC, or PCR. Additionally, heterogeneity in sample sources, patient populations, and study designs—such as the inclusion or exclusion of control groups—likely contribute to conflicting results. Importantly, geographic and demographic factors, as well as differing levels of latent HCMV infection within populations, may also influence detection rates. These inconsistencies highlight the need for standardized protocols and larger, multicenter studies to resolve ambiguities and establish a clearer understanding of the association between HCMV and BC.

**Table 1:** HCMV positivity in BC versus control groups determined by PCR.

Author, Year	Country	Tissue Type	Methods	Region/Gene	HCMV Pos/Total (%)	Comments
Tsai et al., 2005 [100]	Taiwan	Frozen	PCR	IE2	Control = 8/12 (66.7) Fibroadenoma = 20/32 (62.5) IDC = 47/62 (75.8)	Control group included normal, fibrosis, and breast fibrocystic changes. *p* = ns (control vs. fibroadenoma or cases)
Eghbali et al., 2012 [103]	Iran	FFPE	PCR	DNA	Control = 0/24 BC = 2/24 (8.3)	Fibroadenoma samples was used as control. *p* = ns (control vs. cases)
Shadood et al., 2018 [101]	Iraq	FFPE	PCR	DNA	Control = 1/20 (5.0) BC = 5/80 (6.25)	*p* = ns (control vs. cases)
Sepahvand et al., 2019 [91]	Iran	FFPE	PCR	gB	Fibroadenoma = 10/35 (28.6) DCIS = 4/13 (30.8) IDC = 16/24 (66.7)	*p* = 0.028 (Fibroadenoma vs. DCIS + IDC) and *p* = 0.028 (DCIS vs. IDC). HCMV infection was higher in grade III (72.2%) compared to grade II (36.8%) (*p* = 0.044).
Richardson et al., 2015 [105]	New Zealand	Frozen	qPCR	pp65	Control = 2/70 (2.3) IDC = 0/70	-
Nakhaie et al., 2021 [109]	Iran	Frozen	Nested PCR	DNA	Control = 1/49 (2.0) BC = 8/49 (16.3)	Adjacent non-cancerous tissue was used as control group *p* = 0.01 (control vs. cases)
Utrera-Barillas et al., 2013 [102]	Mexico	FFPE	PCR	UL122/UL83	Fibroadenoma = 0/20 BC = 2/27 (7.4)	*p* = ns (Fibroadenoma vs. BC) HCMV was not associated with BC progression
Bakhtiyrizadeh et al., 2017 [104]	Iran	FFPE	RT-PCR	gB	Control = 2/150 (1.3) BC = 0/150	Fibroadenomas, fibrocystic disease and adenosis tissues were used as control group. *p* = ns (control vs. cases)
Mohammed et al., 2015 [110]	Iraq	Frozen	RT-PCR	UL54	Control = 0/30 IDC = 9/38 (23.7)	*p* = 0.003 (control vs. cases)
Khasawneh et al., 2024 [92]	Jordan	FFPE	RT-PCR	gB	Control = 0/30 BC = 15/110 (13.6)	Non-malignant breast lesions were used as control group. *p* = ns (control vs. cases) HCMV infection was associated with grade I BC (*p* = 0.015).

Legend: BC, Breast cancer; IDC, infiltrating ductal carcinoma; DCIS, Ductal carcinoma in situ; FFPE, Formalin-fixed and paraffin-embedded tissues; ns, non-significant.

**Table 2:** HCMV positivity between BC and control groups using IHC and ISH.

Author, Year	Country	Tissue Type	Methods	Region/Protein	HCMV Pos/Total (%)	Comments
Al-Nuaimi et al., 2019 [94]	Iraq	FFPE	IHC	EA	Control = 2/10 (20.0) BC = 56/60 (93.3)	Normal breast tissues were used as control. EA: *p* < 0.01 (control vs. cases) LA: *p* = ns (control vs. cases) EA and LA correlated with HER2 expression (*p* < 0.05) and (*p* < 0.05), respectively.
				LA	Control = 7/10 (70.0) BC = 37/60 (61.7)	
Harkins et al., 2010 [87]	USA	FFPE	IHC	IE1/2	Control = 17/27 (63.0) BC = 32 (96.9)	Non-neoplastic tissue was used as control. IE1/2: *p* = 0.0009 (control vs. cases) E/LA: *p* = 0.0001 (control vs. cases) LA: *p* = ns (control vs. cases)
				E/LA	Control = 6/28 (21.4) BC = 25 (84.0)	
				LA	Control = 11/28 (39.3) BC = 27 (55.6)	
El Shazly et al., 2018 [95]	Egypt	FFPE	IHC	E/IE	Control = 0/61 Fibroadenoma = 0/20 BC = 21/61 (34.4)	Adjacent non-tumor tissues were used as control. *p* < 0.001 for both (control vs. BC) and (Fibroadenoma vs. BC) E/IE expression correlated with ER positivity in BC
				pp65	Control = 15/61(24.6) Fibroadenoma = 4/20 (20.0) BC = 49/61 (80.3)	Adjacent non-tumor tissues were used as control. *p* = 0.005 (control vs. BC) and *p* = 0.003 (Fibroadenoma vs. BC) PP65 expression correlated with HER2 negativity, ER positivity (*p* = 0.004), and RP positivity in BC.
Rahbar et al., 2017 [111]	Norway	FFPE	IHC	IE	Control = 42/42 (100) DCIS = 18/18 (100) BC = 53/53 (100)	Adjacent normal breast tissue was used as control. *p* = ns (control vs. cases) for both molecules. High levels of IE were increased in BC compared to control (*p* < 0.001), and it was related with low levels of ERα expression in BC samples. High levels (>50% cells) of LA were increased in BC compared to DCIS (*p* = 0.01).
				LA	Control = 27/40 (67.5) DCIS = 9/19 (47.4) BC = 46/61 (75.4)	
Mohammed et al., 2015 [97]	Iraq	FFPE	IHC	IE1	Control = 3/30 (10.0) IDC = 34/38 (89.5)	Tissues from safe surgical margin were used as control. IE1 (RR 8.9, 95% CI: 3.04–26.33; *p* = 0.0001) LA (RR 56.35, 95% CI: 3.68–83.84; *p* = 0.0001) pp65 (RR 54.84, 95% CI: 3.58–59.44; *p* = 0.0001)
				LA	Control = 0/30 IDC = 35/38 (92.1)	
				pp65	Control = 0/30 IDC = 34/38 (89.5)	
Cui et al., 2018 [96]	China	FFPE	IHC	IE	Control = 3/40 (7.5) Paracancerous = 70/146 (47.9) BC = 146/146 (100)	*p* < 0.001, for both molecules (paracancerous vs. cases) IE and LA proteins were increased in lymph node metastasis (92.6%) compared to non-metastatic SLN samples (60.0%) (*p* < 0.001, for both). IE expression was negatively correlated with ER (*p* < 0.05)
				LA	Control = 2/40 (5.0) Paracancerous = 78/146 (53.4) BC = 146/146 (100)	
Costa et al., 2019 [93]	Norway	FFPE	IHC	IE	Control = 2/26 (7.7) IDC = 49/49 (100)	Adjacent normal breast tissues were used as control. HCMV IE protein expression was correlated with extensive COX-2 (*p *< 0.0001) or 5-LO (*p * = 0.0003) in BC tissue specimens.
				LA	Control = 0/26 IDC = 11/49 (22.4)	
Ali et al., 2018 [89]	Iraq	FFPE	ISH	DNA	Control = 0/15 Benign tumors = 6/25 (24.0) BC = 17/30 (56.7)	Apparently healthy breast tissues were used as control. *p* < 0.0001 (control vs. benign tumors) *p* < 0.0001 (control vs. BC)
Alajeely et al., 2019 [90]	Iraq	FFPE	ISH	DNA	Control = 7/40 (17.5) BC = 16/40 (40.0)	Samples of benign breast tumors and apparently normal breast tissues were used as control. *p* = 0.041 (control vs. cases) HCMV was associated with a decreased CD4 lymphocytes counts (*p* = 0.0419).

Legend: IHC, Immunohistochemistry; ISH, in situ hybridization; BC, Breast cancer; IDC, infiltrating ductal carcinoma; DCIS, Ductal carcinoma in situ, FFPE, Formalin-fixed and paraffin-embedded tissues, ns, non-significant.

#### 2.2.2. HCMV Infection and BC Aggressiveness

Numerous studies have also demonstrated an association between HCMV infection and the aggressive behavior of BC. Rahbar et al. (2017) reported that HCMV DNA and RNA were detected in all BC samples examined, with a higher prevalence of HCMV IE protein in infiltrating ductal carcinoma (IDC) compared to ductal carcinoma in situ (DCIS) and adjacent non-tumor tissues [111]. Additionally, HCMV DNA was more frequently found in IDC cases (66.66%) compared to DCIS (30.76%) (*p* = 0.028) [91]. Fares et al. (2019) reported a higher prevalence of HCMV DNA in inflammatory breast carcinoma (IBC) compared to non-inflammatory BC (78.6% vs. 21.4%; *p* = 0.030), and they noted a significant increase in Grb2 mRNA expression in HCMV-positive IBC samples [112]. Additionally, another study found that HCMV DNA was more frequently detected in grade III tumors than in grades I or II [91]. Similarly, Karimi et al. (2016) found HCMV DNA in 52.0% of BC tissues, with a significantly higher presence in grade II and III tumors compared to grade I (*p* = 0.04) [113].

Consistent with these findings, Ahmed and Yussif (2016) reported a significant association between HCMV expression and high tumor grade, elevated mitotic count, IDC histology, and specific molecular subtypes [99]. This study also revealed an inverse correlation between HCMV expression and hormone receptor (ER, PR) and HER2 expression [99]. Similarly, Rahbar et al. (2017) [111] and Cui et al. (2018) [96] observed a negative correlation between HCMV IE protein expression and ER and PR expression. However, some studies have reported contradictory findings suggesting no association between HCMV and BC diagnosis [114] nor any potential positive association between HCMV expression and hormone receptor positivity [94].

HCMV infection has also been linked to the expression of key oncogenic factors and the activation of signaling pathways involved in tumor progression. Kumar et al. (2018) identified HCMV lncRNA4.9 in BC samples [115], a gene crucial for viral DNA replication regulation [116]. In IBC samples with HCMV infection, NF-κB/p65 and phospho-NF-κB p65 were significantly higher compared to HCMV-negative samples [117]. High HCMV IE protein expression was also correlated with elevated levels of cyclooxygenase-2 (Cox-2) and 5-lipoxygenase (5-LO) in BC [93]. Additionally, HCMV IE2 mRNA was found in metastatic sentinel lymph nodes (SLN) at significantly higher levels than in non-metastatic SLN samples [96]. Taher et al. (2013) reported HCMV IE expression in 100% of primary BC and observed significantly higher levels in SLN-positive versus SLN-negative cases [118].

The impact of HCMV infection on patient survival has also been investigated [119]. Touma et al. (2023) reported that HCMV IE positivity was associated with reduced median overall survival (OS) in BC patients compared to those with IE-negative tumors (118.4 vs. 202.4 months; *p* = 0.04). Patients with tumors containing ≥ 25% LA-positive cells had a shorter OS than those with < 25% LA-positive cells (146.2 vs. 151.5 months; *p* = 0.04) [120]. Additionally, HCMV DNA presence in breast tumors was associated with shorter relapse-free survival (*p* = 0.042) [121].

These findings collectively suggest that HCMV infection may contribute to a more aggressive BC phenotype, particularly in higher-grade tumors and invasive breast carcinoma subtypes. The observed associations between HCMV protein and RNA expression and key markers of tumor aggressiveness underscore the virus’s potential role in promoting BC progression.

## 3. Potential Mechanisms of HCMV-Induced BC

Certain HCMV strains can infect breast epithelial cells, with high-risk strains (e.g., HCMV-DB and BL) more effectively activating oncogenic pathways in infected mammary cells than low-risk strains (e.g., HCMV-FS, KM, and SC) [115,122,123]. For instance, the clinical HCMV-DB strain can transform human mammary epithelial cells (HMECs) after prolonged culture, leading to the development of CMV-transformed HMECs (CTH), which exhibit various oncogenic characteristics [115,124]. Notably, CTH cells can undergo giant cell cycling, producing polyploid giant cancer cells (PGCC), which play a significant role in the transformation of breast epithelial cells by HR-HCMV strains [125]. The potential mechanisms by which HCMV may contribute to breast carcinogenesis are outlined in the following sections.

### 3.1. Activation of Signaling Pathways That Control Cell Proliferation

HCMV infection can significantly disrupt normal cellular processes, leading to uncontrolled cell proliferation. In HMECs infected with the HCMV-DB strain, the expression of several oncogenes, including *Myc*, *Fos*, *Jun*, and *Ras*, is markedly upregulated, promoting cell proliferation [126]. Furthermore, HCMV-DB infection activates the signal transducer and activator of transcription 3 (STAT3) in HMECs, leading to the upregulation of cyclin-D1, a critical regulator of cell proliferation, and a marked increase in Ki-67 protein expression in infected cells [115]. An elevated activation of AKT and STAT3, along with upregulated cyclin D1 and Ki-67, has also been observed in HMECs infected with clinical HCMV strains [127].

The HCMV gene *UL111A* encodes cmvIL-10, a viral homolog of the human immunomodulatory interleukin-10 (IL-10), which is secreted by infected cells [128,129]. CmvIL-10 binds to IL-10 receptors (IL-10R), stimulating the proliferation, migration, and invasion of BC cells [130,131]. Treatment with exogenous cmvIL-10 in BC cells activates the Janus kinase 1 (JAK1)/STAT3 pathway, leading to increased cell proliferation [132]. The activation of *c-Myc* and *Ras* proto-oncogenes in HMECs infected with HCMV-DB has also been linked to enhanced activity in the phosphoinositide 3-kinase (PI3K)/Akt pathway [115]. HCMV infection in BC samples correlates with an increased mitotic count, further supporting its role in promoting proliferation [99].

HCMV infection can also impair cell cycle regulation by targeting tumor suppressor proteins. The HCMV IE2 protein binds to p53, inhibiting its function and preventing the induction of cell cycle arrest. Furthermore, HCMV infection leads to the hyperphosphorylation and downregulation of RB, further compromising cell cycle control [115].

Collectively, these studies highlight the ability of HCMV to manipulate key signaling pathways involved in cell proliferation, which could contribute to the development and progression of BC.

### 3.2. Regulation of Apoptosis and Cell Survival

HCMV-encoded proteins have been shown to disrupt apoptotic pathways and promote cell survival. For instance, cmvIL-10 has been shown to protect cells from apoptosis induced by etoposide, a topoisomerase II inhibitor commonly used in cancer therapy [132]. Transcriptomic analysis of HMECs infected with the HCMV-DB strain demonstrated a downregulation of the caspase-8 (*CASP8*) transcript, an essential upstream mediator of apoptosis. Moreover, there was an upregulation of Bcl-2, a prominent anti-apoptotic protein that prevents mitochondrial-mediated cell death [126]. In addition to Bcl-2, several key genes involved in cell survival and proliferation pathways were upregulated, including *AKT1* and *PIK3C2A*, which are integral to the PI3K/AKT pathway, a major signaling pathway that promotes cell growth and inhibits apoptosis. The increased expression of *NFKB1* and *REL*, key components of the NF-κB pathway, further emphasizes the role of HCMV in fostering a pro-survival environment within infected cells. NF-κB activation not only inhibits apoptosis but also promotes inflammatory responses, potentially contributing to a tumor-promoting microenvironment. These alterations suggest that HCMV infection could enable cells to evade programmed cell death, enhancing their survival and potentially contributing to oncogenic transformation and tumor progression.

### 3.3. Induction of Epithelial-to-Mesenchymal Transition (EMT) and Cell Migration

EMT is a cellular reprogramming process in which epithelial cells acquire mesenchymal traits, resulting in enhanced motility, invasiveness, and resistance to apoptosis [133]. EMT is characterized by the downregulation of epithelial markers like E-cadherin and the upregulation of mesenchymal markers such as N-cadherin and vimentin [134], contributing to a more migratory and invasive cell phenotype. While EMT plays an essential role in development and wound healing, it is often hijacked by cancer cells to promote metastasis, enabling them to detach from primary tumors, invade surrounding tissues, and establish secondary growths [135].

**Figure 1:**
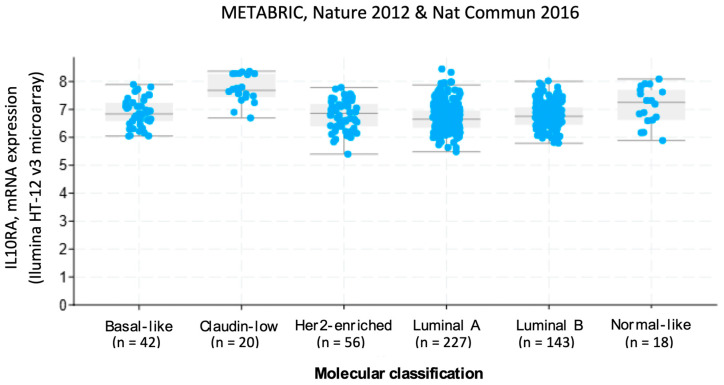
Levels of IL10RA in primary breast primary tumors according to the molecular classification (METABRIC, Nature 2012 & Nat Commun 2016 [137], groups from Pam50 + Claudin-low subtype, n = 548). An increased expression of IL10RA mRNA was evidenced in claudin-low BC compared to the other molecular subtypes (*p* = 1.89 × 10^−15^, one-way ANOVA). Raw data were extracted from the cBioPortal for Cancer Genomics, Memorial Sloan Kettering Cancer Center (MSK) (https://www.cbioportal.org, accessed on 29 October 2024) [142,143,144].

In studies involving tumor samples from mice injected with HCMV-infected BC cells (CTH), EMT-associated changes were observed, including a decrease in E-cadherin and an upregulation of vimentin, suggesting that HCMV infection promotes EMT in these cells [115]. The treatment of BC cells with cmvIL-10 led to a 2.24-fold increase in the expression of *FXYD5*, an ion transport regulator linked to decreased cell adhesion and increased invasiveness [131,136]. According to METABRIC, Nature 2012 & Nat Commun 2016 cohorts [137], the expression of IL-10 receptor A (IL10RA) was higher in claudin-low breast carcinomas compared to the other molecular subtypes. These tumors are characterized by the suppression of some epithelial cell adhesion molecules and EMT features [138] (Figure 1).

CmvIL-10 treatment also led to elevated levels of CD146, or melanoma cell adhesion molecule, in BC cells [131]. CD146, or melanoma cell adhesion molecule, is known to promote the loss of cell–cell contacts, EMT, and enhanced cell motility, contributing to the invasive potential of cancer cells [139,140]. In addition, cmvIL-10 upregulated the expression of matrix metalloproteinases (MMPs) such as MMP-3, MMP-9, and MMP-10, while downregulating metastasis suppressor 1 (MTSS1) in BC cells, which may facilitate tumor cell dissemination and increase metastatic potential [130,131].

CmvIL-10 also increased the expression of the urokinase plasminogen activator receptor (uPAR) in BC cells [131]. The uPA/uPAR system plays a role in degrading the extracellular matrix, a process necessary for vascular endothelial growth factor (VEGF)-mediated angiogenesis [141]. In BC cells expressing EGFR, cmvIL-10 enhanced cell migration in the presence of EGF, although cmvIL-10 alone did not stimulate cell movement [132].

### 3.4. Promotion of Cell Immortalization and Tumor Formation

Cellular immortalization, the capacity for indefinite replication, is a pivotal step in the evolution of malignant tumors. A primary mechanism behind this is the upregulation of human telomerase reverse transcriptase (hTERT), an enzyme that sustains telomere length and prevents cellular senescence [145]. In recent studies, HMECs infected with the HCMV-DB strain exhibited increased *hTERT* mRNA levels and telomerase activity compared to uninfected cells [115]. This increase could allow HCMV-infected cells to bypass replicative senescence caused by telomere shortening, enabling sustained proliferation [146]. Additionally, HMECs infected with HCMV-DB, but not the AD169 strain (a commonly used laboratory strain of HCMV) [147], demonstrated anchorage-independent growth, a characteristic of transformed cells [115]. Colony formation in soft agar was also observed in HMECs infected with HCMV strains B544 and B693, both isolated from TNBC cases with a high expression of enhancer of zeste homolog 2 (*EZH2*) and *Myc* [124]. EZH2, a histone methyltransferase, is linked to aggressive behavior in several cancers, including TNBC [148].

Additional tumor-promoting effects were observed with the cmvIL-10 treatment of BC cells. This viral cytokine downregulated tumor suppressor and cell adhesion genes, including spleen tyrosine kinase (*SYK*), adenomatous polyposis coli (*APC*), and *CDH1* [132]. Specifically, *SYK* knockdown has been shown to enhance proliferation, invasion, and anchorage-independent growth in BC cells, further facilitating tumor progression [149].

HCMV-DB-infected cultures also displayed an elevated proportion of stem-like cells capable of forming tumorspheres, a feature absent in uninfected cells [126]. The tumorigenic capacity of these transformed cells was confirmed in vivo: clusters of HCMV-DB-transformed cells (CTH) induced tumor formation in NOD/SCID Gamma (NSG) mice, a result not observed with uninfected cells [115]. These findings underscore the impact of HCMV infection and cmvIL-10 on enhancing cellular immortalization and tumor formation, thereby highlighting the virus potential role in cancer progression.

### 3.5. Induction of a More Aggressive Tumor Phenotype and Stemness

HCMV infection has been shown to drive a more aggressive and stem-like phenotype in BC cells, enhancing their tumorigenic potential and metastatic capacity. Transcriptomic analysis of HCMV-DB-infected human mammary epithelial cells (HMECs) revealed a shift towards a triple-negative basal-like phenotype, characterized by ER-/PR-/HER2- status and a reduced expression of luminal markers, including GATA3, *TFF1*, KRT18, and KRT19 [126]. Immunohistochemical analysis of tumor tissues from mice injected with HCMV-transformed cells (CTH) confirmed this triple-negative profile, showing ER-/PR-/HER2- status and an absence of proteins such as CK5/6, GATA3, CK20, and GCDFP [115]. Furthermore, HMECs infected with HR-HCMV strains exhibited higher expression levels of the oncogenes *Myc* and *EZH2* compared to cells infected with low-risk strains, underscoring the aggressive potential conferred by HCMV [124].

In addition to fostering a more aggressive phenotype, HCMV-DB infection appears to enhance cellular stemness. Stemness markers, including Oct4, Tra-1-60, SOX2, Nanog, and SEA-4, are key indicators of a cell’s ability to self-renew and differentiate, characteristics commonly associated with cancer progression and resistance to treatment [150]. These markers were significantly upregulated in CTH cells compared to uninfected HMECs. An increase in the expression of CD49f, CD44, and CD24 was also observed in polyploid giant cancer cells (PGCCs) relative to smaller cells [124]. An elevated CD44/CD24 ratio, typically associated with basal-like BC, correlates with increased proliferation and tumorigenic capacity in BC cells [151,152]. Moreover, the presence of PGCCs was associated with lymph node metastases in BC, suggesting a link between these cells and disease progression [153]. These findings highlight the role of HCMV in promoting a highly aggressive, stem-like phenotype in BC, contributing to enhanced proliferation, tumorigenicity, and metastatic potential.

### 3.6. Influence of HCMV on the Tumor Microenvironment (TME) and Immune Response

The TME comprises malignant tumor cells, endothelial cells, immune cells, various stromal cells, and extracellular matrix components [154]. This complex environment plays a pivotal role in multiple aspects of tumorigenesis, including the formation of tumor vasculature, which is crucial for metastasis progression and therapeutic responses [155]. HCMV significantly influences the TME by altering both its cellular and molecular components, thereby contributing to oncogenesis and tumor progression [156]. Notably, HR-HCMV strains, such as HCMV-DB, exhibit a strong tropism for macrophages, inducing a shift from pro-inflammatory M1 macrophages to tumor-promoting M2/TAM phenotypes. This macrophage polarization enhances tumor progression by impairing innate immunity, remodeling tissue architecture, and promoting angiogenesis through the upregulation of VEGF [157].

Additionally, HCMV employs multiple mechanisms to evade immune surveillance, including the suppression of CD4^+^ and CD8^+^ T-cell responses and natural killer (NK) cell activity [158]. This immune evasion is achieved through the inhibition of antigen presentation, the production of immunosuppressive cytokines such as viral IL-10 (CmvIL-10), transforming growth factor-beta (TGF-β), and the modulation of interactions between T-cell and NK cell receptors [159,160]. These strategies foster an immunosuppressive environment characterized by increased regulatory T cells (Tregs), CD28^−^ T cells, and Th17 cells, collectively promoting viral persistence, chronic inflammation, tumor growth, and relapse [161].

**Figure 2:**
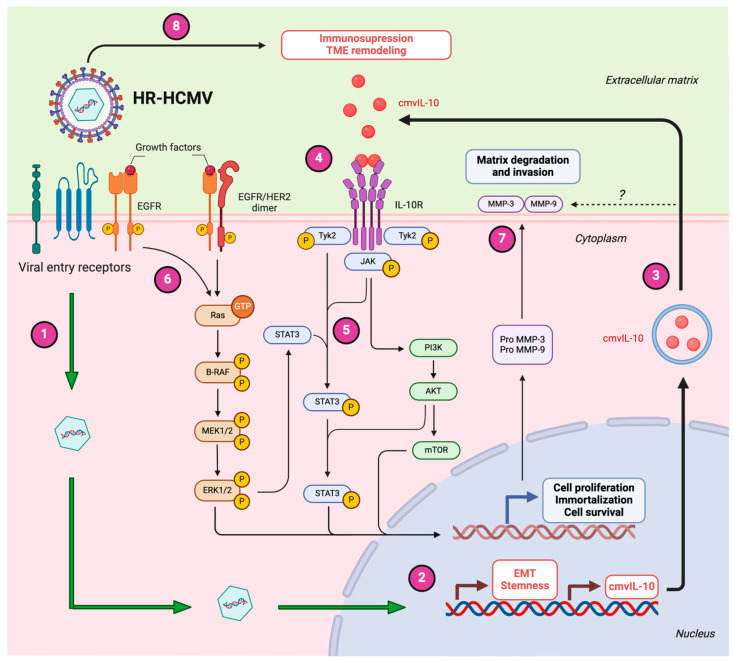
A proposed model of HCMV’s contribution to BC development. 1: HR-HCMV enters into breast epithelial cells, and viral DNA is transported inside the capsid and released into the nucleus. 2: The expression of HCMV proteins may induce epithelial-to-mesenchymal transition and also activate stemness pathways. 3: In particular, cmvIL-10 is secreted in vesicles by infected cells, exerting its immunomodulatory properties. 4: The cmvIL-10 dimers could also bind and stimulate the IL-10/IL-10 receptor (IL-10R) axis. 5: The activation of oncogenic pathways such as JAK/STAT and PI3K/AKT signaling provokes cell proliferation, immortalization, and cell survival. 6: The EGFR/RAS/RAF/MAPK oncogenic pathway could be activated during HCMV entry into breast epithelial cells. 7: The cmvIL-10 could increase the activity of matrix metalloproteinases (e.g., MMP-3, MMP9), which in turn induce matrix degradation and invasion. 8: The release of HR-HCMV viral particles modulates the tumor microenvironment (TME), promoting immune evasion and creating favorable conditions for tumor progression, invasion, and the survival of infected breast epithelial cells. The question mark (?) indicates that the potential mechanisms by which cmvIL-10 could increase the activity of MMPs need further investigation. Created by BioRender.com.

Overall, these findings emphasize the role of HCMV in molding the TME to establish a conducive environment for tumor advancement and in altering the immune response. A hypothetical model depicting the potential role of HR-HCMV strains in BC development is illustrated in Figure 2.

## 4. Conclusions

The potential role of HCMV in BC carcinogenesis continues to be a subject of significant interest. This review has examined the epidemiological evidence linking HCMV infection to BC and explored the molecular mechanisms by which HCMV may contribute to BC initiation and progression.

The available literature suggests that HCMV could play a multifaceted role in breast carcinogenesis. The direct infection of epithelial cells may lead to cellular transformation and tumorigenesis. Additionally, the HCMV infection of non-epithelial cells, such as immune cells, may create a tumor-supportive microenvironment that promotes abnormal epithelial cell growth. Geisler et al. (2019) have highlighted the potential immunosuppressive effects of HCMV in BC [162]. Certain HCMV strains with strong oncogenic properties have been shown to induce tumor formation in animal models and may be associated with more aggressive BC subtypes, such as TNBC.

To further elucidate the role of HCMV in BC, future research should focus on sequencing HCMV strains in BC samples. Identifying high-risk strains could provide valuable insights into strain-specific mechanisms driving BC progression, potentially enabling the characterization of disease trajectories. Such efforts could lead to the identification of novel biomarkers for predicting disease progression, therapeutic response, and overall survival outcomes in BC patients. These biomarkers could help refine personalized treatment strategies and improve long-term prognosis. However, additional clinical and molecular studies are necessary to validate the clinical significance of HCMV in BC and translate these findings into actionable clinical interventions.

## Data Availability

Not applicable.
